# AIDS-related mortality in Pará Province, Brazilian Amazon region: Spatial and temporal analysis

**DOI:** 10.1371/journal.pone.0279483

**Published:** 2023-01-20

**Authors:** Taymara Barbosa Rodrigues, Bruna Rafaela Leite Dias, Dulce Gomes, Ricardo Alexandre Arcêncio, Jorge Alberto Azevedo Andrade, Glenda Roberta Oliveira Naiff Ferreira, Lucia Hisako Takase Gonçalves, Eliã Pinheiro Botelho

**Affiliations:** 1 Faculty of Nursing, Institute of Health Sciences, Federal University of Pará, Pará, Brazil; 2 Department of Mathematics, University of Évora, Évora, Portugal; 3 Department of Maternal-Infant Nursing and Public Health, University of São Paulo at Ribeirão Preto College of Nursing, Ribeirão Preto, Brazil; 4 Department of Epidemiology and Health Surveillance, Evandro Chagas Institute, Pará, Brazil; National Taiwan University, TAIWAN

## Abstract

Despite considerable therapeutic advances in the care of people living with human immunodeficiency virus (HIV) and with the acquired immunodeficiency syndrome (AIDS) and an overall reduction of 47% in the AIDS mortality rate in the last decade, the AIDS-mortality rates remains high. The social determinants of health (SDH) have a direct influence on the dynamics of this phenomenon. However, changes in SDH caused by the implemented policies against HIV have been poorly investigated. Moreover, the Brazilian rainforest has had the highest and continuously increasing AIDS mortality rate in Brazil since the 1980s. In this study, AIDS mortality in a province of the Brazilian rainforest was examined by using temporal and spatial analyses. **Methods.** In this ecological study, data from 2007 to 2018 were extracted from the Mortality Information System provided by the State Department of Public Health of Pará. For the temporal analysis, the integrated autoregressive model of moving average (ARIMA) and locally weighted polynomial regression (STLF) were used to forecast AIDS mortality from 2019 to 2022. For the spatial analysis, spatial autocorrelation and geographically weighted regression (GWR) analyses were employed. **Results.** The samples consisted of 6,498 notifications for AIDS-related deaths. From 2007 to 2013, the AIDS mortality rates showed an upward trend, followed by a stabilization until 2018 and an upward forecasted trend from 2019 to 2022. High mortality rates and high-high clusters were found in economic pole municipalities. Furthermore, AIDS mortality risk was directly associated with per capita income and demographic density, except in the southwestern region of Pará, which exhibited an inverse association with population density. **Conclusion.** Although the policies against HIV may have contributed to the stabilization of AIDS mortality rates from 2013 in Pará, the upward forecasted trend until 2022 raises an alert and concern to health authorities to provide reinforcement of the policies. The geographic variability of AIDS mortality promoted by SDH provides subsidies to health authorities to implement SDH-focused strategies for AIDS mortality reduction.

## Introduction

Despite considerable therapeutic advances in the care of people living with HIV and AIDS (PLWHA) and an overall reduction of 47% in the AIDS mortality rate in the last decade, the incidence of this disease remains high. In 2020 alone, 680,000 AIDS-related deaths were reported worldwide [1]. In the last decade, the AIDS mortality rate in Brazil decreased by 29.1% (2009: 5.8; 2019: 4.1; x100,000 inhabitants); nevertheless, it still ranked as the 14th cause of mortality in the country worldwide and 1st in Latin America with the highest mortality rate [2,3]. In Brazil, the Amazon region has been a serious problem to Brazilian health authorities on reducing the Brazilian AIDS mortality. Although all public Brazilian policies to fight HIV, the Amazon region still experiencing a continuous increasing of the AIDS mortality rates since 1980s with an increase of 18.52% in the rate only from 2009 to 2019 [2].

The fight against HIV (with a consequent reduction in the AIDS mortality rate) involves the pursuit of social equality. An analysis of the social determinants of health (SDH) and their relationship with AIDS mortality can explain how the living and working conditions of individuals and population groups affect their health statuses in different scenarios [4,5].

Ecological studies constitute a field of research that allows for the analysis of such associations through the aggregation of spatial and temporal tools capable of deciphering how the construction of the territory interferes with the social determinants of health. In addition, they also allow for the identification of patterns capable of predicting results and for recognizing the influences of the SDH on the problem that is being studied [6–8].

By employing the descriptors “AIDS-mortality” and “ecological study” and “spatial analysis” or “temporal analysis”, we noticed that there is a scarcity of studies employing spatial [9–13] and temporal analyses [11,12,14,15]. Only one study employed geographic weighted regression (GWR) to analyze the SDH influences on the spatial variability of the AIDS mortality rate [13] with the other studies only making inferences on SDH influences. Additionally, the temporal studies were restricted to the annual variation of the AIDS-mortality rates, thus excluding other important time series aspects, such as seasonality, breakpoint change and time series forecasting. These facts demonstrate the necessity of more robust analysis techniques to study AIDS mortality.

While the breakpoint analysis shows the exact point when a time series changes behavior, the seasonality analysis shows the time periods wherein the evaluated problems are increased or decreased. Additionally, the forecasting analysis provides estimates of the direction of future trends of the studied problem. Associated, both temporal and spatial analyses are essential tools for evaluation of public policies show territorial areas needing more attentions.

Therefore, the main goal of this study was to analyze AIDS mortality in Pará by employing spatial and temporal analyses. In the spatial analysis, we employed the spatial distribution and spatial autocorrelation of global Moran’s I and spatial scan to identify the spatial and spatiotemporal risk areas for AIDS mortality and GWR to examine how and which SDH factors were influencing the spatial variability in AIDS mortality. For the temporal analysis, we used the breakpoint function and a robust hybrid temporal technique, which comprises the integrated autoregressive model of moving average (ARIMA) and locally weighted polynomial regression (STLF), to analyze seasonality and for forecasting the AIDS mortality rate.

This is the first study to analyze AIDS mortality in Pará, Brazil, which is a part of the Brazilian Amazon region. This state has had the highest and continuously increasing AIDS mortality rate since the 1980s. Pará and its capital (Belém) are ranked as the second state and capital, respectively, with the highest AIDS mortality rates. From 2009 to 2019, AIDS mortality in Pará increased by 26.2% (2009: 6.1, 2019: 7.7/100,000 inhabitants) [2]. This study provides strong subsidies to health authorities for implementing more efficient public policies to decrease AIDS mortality in Northern Brazil.

## Materials and methods

### Study setting and design

**Fig 1:**
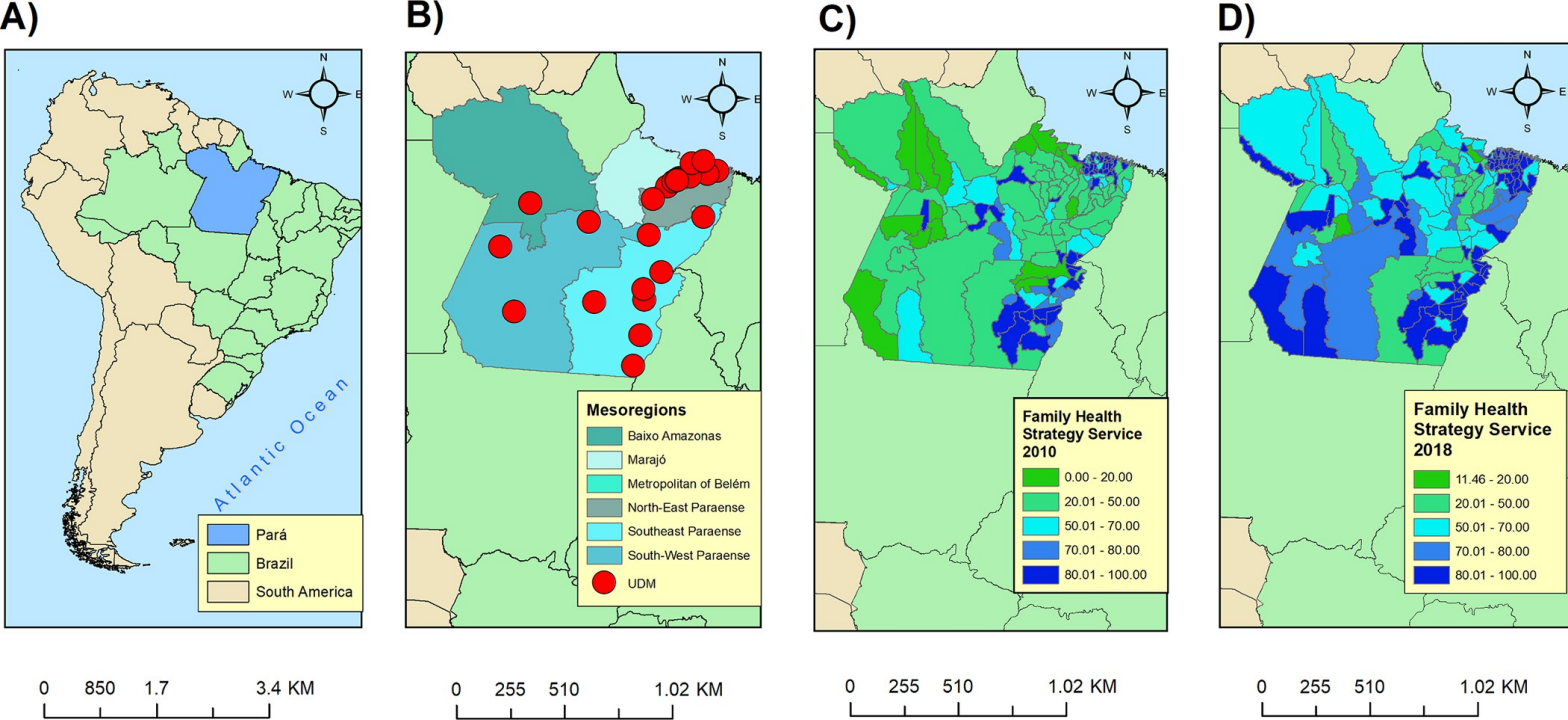
Location map of the study setting, State of Pará, Brazil. (A) Map of the South American continent. Brazil is highlighted in green and Pará in blue; (B) Mesoregions of Pará and punctual spatial location of the Medications Dispensing Units; (C) The Family Health Strategy Services coverage in all municipalities of Pará in 2010, and (D) in 2018. Source: Authors.

This ecological study was conducted in the state of Pará, which is located in the northern region of Brazil (Fig 1). Pará is the second largest state in Brazil, with an area of 1,245,759.305 km^2^, and is bordered by Suriname and Amapá to the north, the Atlantic Ocean to the northeast, Maranhão to the east, Tocantins to the southeast, Mato Grosso to the south, Amazonas to the west, and Roraima and Guyana to the northwest. Pará has 144 municipalities that are grouped into six political mesoregions (Fig 1B): the Baixo Amazonas, Southwest, Marajó, Northeast, Metropolitan, and Southeast [16].

Pará has an estimated population of 8,513,497 inhabitants, approximately 31% of whom are composed of riverside dwellers, maroons, indigents, and other people living in rural zones [16,17]. In addition to geographic barriers such as densely forested areas and rainy weather, the state of Pará has the third lowest Human Development Index in Brazil (0.646) [18]. With regard to policies against AIDS, Pará has only 28 medication dispensing units (MDU) for anti-retroviral drugs (Fig 1B), and 7 specialized HIV/AIDS health care facilities [2,19]. And, spite of the primary health care coverage expansion between 2010 and 2018 (Fig 1C and 1D, respectively), Pará has only 59.13% of primary healthcare services coverage.

### Data sources

All the data were extracted from the Mortality Information System (SIM) provided by the State Department of Public Health of Pará. All of the notifications of AIDS-related deaths with home addresses in Pará between 2007 and 2018 were included. The following variables were collected: municipality of residency, data of the death notification, sex, age, and race/skin color.

SDHs were extracted from the database of the Brazilian Institute of Geography and Statistics based on the 2010 population censuses; the 2020 demographic census in Brazil had not been completed as of11/30/2021. According to the previous literature review, the most frequent SDH was selected and categorized into five dimensions: **Human Development** (Municipal Human Development Index), **Income and Work** (GINI Index, average per capita income, proportion of vulnerable to poverty, and unemployment rate of the population aged 18 years or older), **Education** (illiteracy rate of the population aged 18 years or older and expectation of years of schooling at 18 years of age), **Demography** (proportion of rural population and population density), and **Health care coverage** (family health strategy coverage). Information about the coverage of family health strategies was extracted from the Brazilian Primary Care Information and Management System (E-Gestor AB) [20].

### Statistical analysis

#### Temporal analysis

The monthly crude AIDS mortality rate was employed in the temporal analysis of the historical series. The mortality rate was calculated by dividing the total number of death notifications in the month by the state projected population in the respective year of the month. The results were then multiplied by 100,000 inhabitants.

Series trends and seasonality were characterized via seasonal and trend decompositions by using the Loess (STL) method developed by Cleveland et al. [21]. Breakpoints of the series were estimated by using the breakpoints function of the R Cran library structure via the examination of the test structural changes based on relationships between linear regressions. The confidence interval of the breaks was obtained using the function *confint* with confidence interval level of 95% [22,23].

Due to the nonstationary attributes of the series, the modeling of mortality rates and the prediction of the respective trends were conducted by using the ARIMA of order *p*, *d*, and *q*, where *p* represents the order of the autoregressive component, *d* is the order of differentiation, and *q* is the order of the moving average component [24]. The ARIMA model encompasses the autoregressive model. the moving average model, or the combination of both. A nonseasonal ARIMA (*p*, *d*, and *q*) model was used and written as:

$$y^{\prime }t=c+\phi 1y^{\prime }t-1+\cdots +\phi py^{\prime }t-p+\cdots +\theta q\varepsilon t-1+\varepsilon t.,$$

where y′t represents a differentiated series; *c* is the mean of the changes between consecutive observations (usually known as a constant); and εt denotes the errors, which is considered white noise (a stationary, uncorrelated, and zero-mean process) [24].

Time series modeling was performed following the stages proposed by Box and Jenkins [25]; specifically, the stages included model identification, parameter estimation, model validation, and prediction of future values.

The model was adjusted using the *auto*.*arima* function of the R Cran library forecast based on the estimation of the maximum likelihood of the model parameters and the choice of the best model based on the criterion of the lowest values of the model. The parameters included the Akaike information criterion (AIC), corrected Akaike information criterion (AICc), and Bayesian information criterion (BIC). Normality (Lilliefors test), constant variance (F test), and the existence of autocorrelation (Box–Pierce test) were used to validate the model in terms of the residuals analysis.

After the best ARIMA model was obtained, the AIDS mortality rate was forecasted from 2019 to 2022 with confidence intervals of 80 and 95%, respectively. These predictions assume that past behavior patterns will be maintained in the future. Although the predictions do not include future events, such as the COVID-19 pandemic, they may demonstrate the impact of these events throughout the study period.

Given that the predictive performance of the adjusted ARIMA model (assessed via the test and training sets) was not satisfactory, the seasonal and trend decompositions using the Loess forecasting (STLF) method was applied in this study [26]. This method combines the STL methodology [21] with ARIMA models to replicate patterns for the future [27].

The predictive performance of the two methods was assessed based on the following measures: root mean square error (RMSE). mean absolute error (MAE), and mean absolute percentage error (MAPE). The most appropriate model had the lowest values for the errors [24].

All of the temporal analyses were performed by using *R Studio® version 1*.*4* software (RStudio, Inc. Washington, DC, USA).

#### Spatial analysis

For the spatial analyses, the AIDS mortality rate was standardized by age to avoid the impact of population variations on the calculations among municipalities employing direct methods. In addition, due to the annual variations, the rates were calculated per four-year time periods (2007–2010, 2011–2014, and 2015–2018) and for the entire time period (2007–2018).

For the spatial autocorrelation analysis, the Moran Global index (*I*) was initially used. *I* represent the degree of spatial autocorrelation of the phenomenon under study, with values ranging from -1 to 1, wherein -1 to 0 indicates inverse correlation, 0 indicates no correlation, and 0 to 1 indicates direct correlation.

The Moran Global index was obtained through the equation:

$$I=\frac{\sum_{i=l}^{n}\sum_{j=l}^{n}w_{ij}(z_{i}-\bar{z})(z_{j}-\bar{z})}{\sum_{i=l}^{n}{\left(z_{i}-\bar{z}\right)}^{2}}$$

where n represents the number of areas, *z*_*i*_ is the value of the attribute considered in area *i*, *z* is the average value of the attribute in the study region, and *w*_*ij*_ represents the elements of the normalized spatial matrix of proximity.

The local indicator of the spatial autocorrelation (LISA) method with 999 permutations was used to visualize the clusters indicated by *I*. In this analysis, clusters were classified as high–high and low–low (direct correlation) and low–high and high–low (inverse correlation) [28]. For both analyses, the queen-type W contiguity matrix was used, and neighboring municipalities were considered as those sharing borders and nodes.

A geographic weighted regression (GWR) was used to identify the relationship between AIDS mortality (the dependent variable) and SDH (the independent variable). GWR is a robust spatial modeling technique used to identify geographically variable relationships and to indicate where the locally weighted regression coefficients diverge from their global values, thus allowing for the spatial visualization of how much the general model explains the phenomenon in each specific area.

First, a Pearson’s correlation was conducted to assess the degree of correlation between the dependent and independent variables. All of the statistically significant Pearson correlation coefficients (*p*<0.05) were then analyzed through the ordinary least squares (OLS) model employing the stepwise method, thus allowing for the testing of different combinations to identify the best explainable regression model. Multicollinearity was examined, and Oonly models with variance inflation factors (VIFs) lower than 10 were considered.

Since the OLS residues were spatially dependents, we applied the Lagrange multiplier test to find an alternative regression model. The best alternative model, spatial lag or the spatial error, was chosen based on statistical significance of LM test [29].

The best model considered for the GWR validation was the model with the lowest AIC, greater R^2^ and adjusted R^2^, smaller VIF, and residuals without spatial dependence according to the Moran’s index [29–31].

Spatial analyses were performed by using the software *Geoda* version 1.14.0 and *ArcMap®* version 10.5 (ESRI, Redland, California, United States).

All the cartographic bases in Shapefile that were used for constructing the thematic maps were obtained by the public database of IBGE [15].

### Ethical aspects

This study is a part of a major project entitled “Situational Diagnosis of Sexually Transmitted Infections in the Amazon Context: Geospatial Analysis, Screening and Development of Educational Care Technologies”, of the National Program for Academic Cooperation (PROCAD), which was approved by the Research Ethics Committee of the Institute of Health Sciences, Federal University of Pará, under Certificate of Presentation for Ethical Appreciation (CAAE) no. 3,331,577.

## Results

During the study period, 6,498 AIDS-related deaths were reported to SIM. The highest crude mortality rate (x100,000 inhabitants) occurred in males (9.5), people aged between 40 and 49 years (16.43), and those individuals with black and brown skin colors (8.11 and 7.73, respectively).

**Fig 2:**
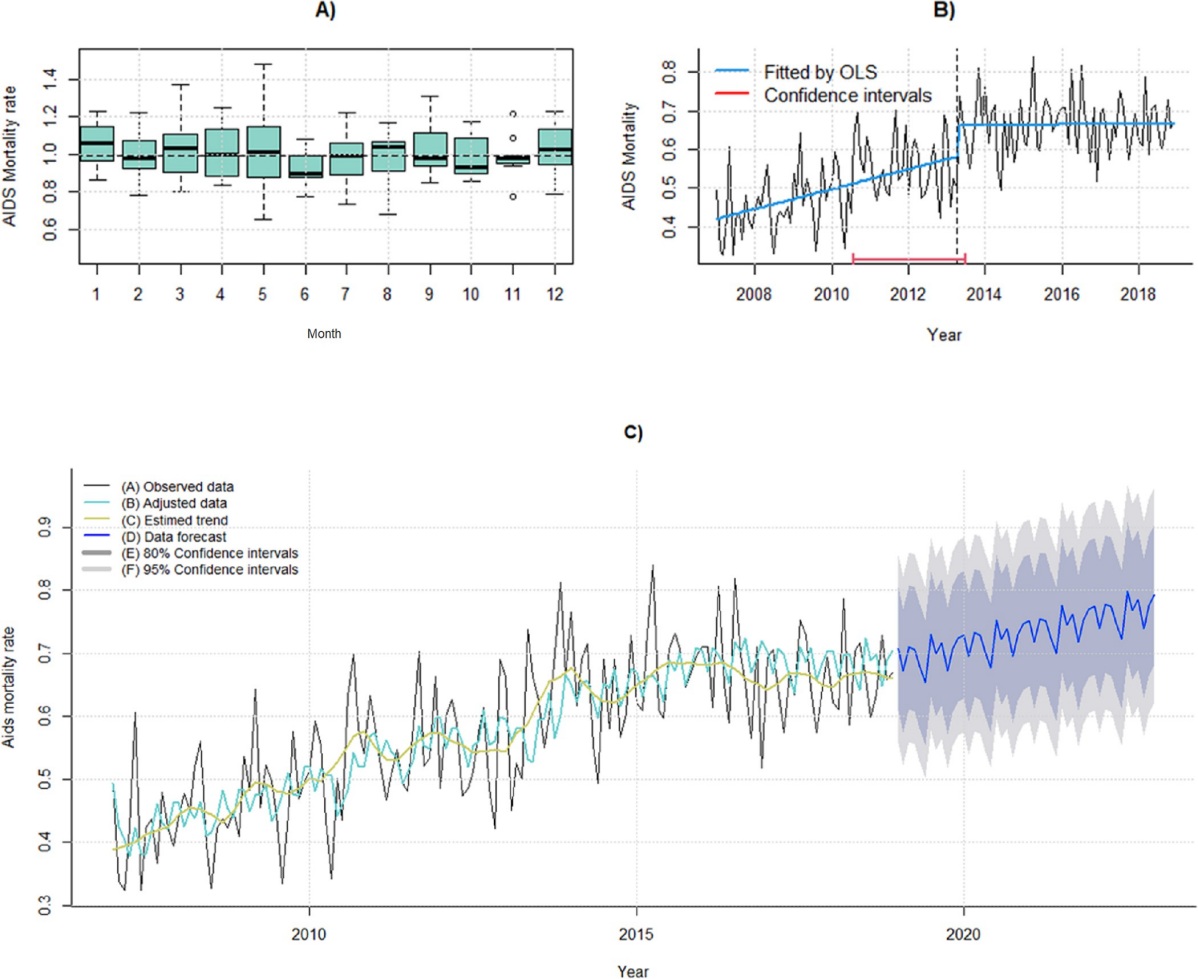
Analysis of the historical series of AIDS mortality rate in the state of Pará from 2007 to 2018. A) Monthly variability of AIDS-mortality rate (standardized by age), B) Variability of the temporal trend of AIDS- mortality rate, and C) Forecast of AIDS-mortality rate in Pará from 2019 to 2022 according to the ARIMA model (0,1,1). Source: Authors.

The historical series analysis of the AIDS crude mortality rate showed no seasonal patterns in the STL decomposition. However, March and May exhibited great variation in the mortality rate (Fig 2A). The breakpoint analysis (CI 95%) showed an increasing trend between January 2007 and April 2013, followed by stabilization until December 2018 (Fig 2B).

The ARIMA model (0,1,1) can accurately describe the variability of data over time, with nonself-correlated residuals, a normal distribution, and constant variance (Table 1). However, for the prediction of the crude mortality rate between 2019 and 2022, the combined STLF and ARIMA models produced the best predictive quality, with a chance of 10.69% of being incorrect (MAPE = 10.69%) (Table 2). Fig 2C shows the model fitted (blue light line) to the original series and its predictions of the increasing AIDS mortality rate from 2019 to 2022.

**Table 1:** Validation tests of the residuals from the ARIMA model (0,1,1) of the series without Box Cox transformation.

Criterion	Test	Statistics	P value
Normality	Lilliefors test	0,050	0,49
Variance	F -Test	1,340	0,32
Autocorrelation	Box-Pierce test	0,905	0,34

**Table 2:** Predictive analysis of mortality rate models.

MODEL	RMSE	MAE	MAPE
ARIMA (0,1,1)	0,084	0,067	17,96
STFL+ ARIMA (0,1,1)	0,075	0,059	10,69

**Fig 3:**
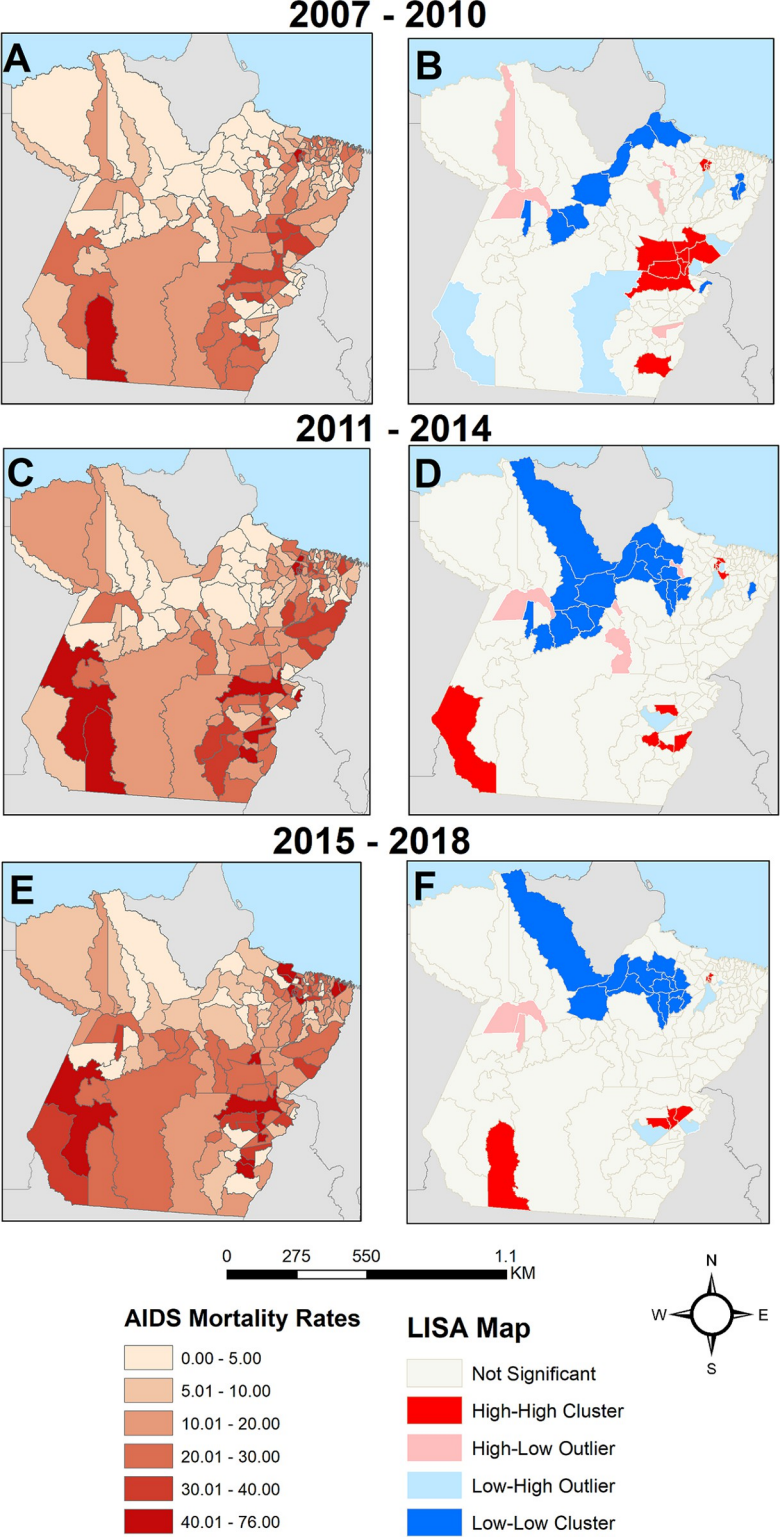
Spatial distribution and local Moran analysis of AIDS mortality rate standardized by age in Pará from 2007 to 2018. A) Spatial distribution for 2007–2010; B) LISA map for 2007–2010, C) Spatial distribution for 2011–2014, D) LISA map for 2011–2014, E) Spatial distribution for 2015–2018, and F) LISA map for 2015–2018. Source: Authors.

The spatial analysis showed geographic variability in the AIDS mortality rate standardized by age in Pará (Fig 3), with an increase over time in the southeast, southwest, and Marajó regions. The spatial autocorrelation analysis of the global Moran index (*I*) showed the existence of spatial dependence in the total period (*I* = 0.372; p≤0.001) and per four-year time periods (2007–2010: *I* = 0.315; *p*≤0.001, 2011–2014: *I* = 0.279; *p*≤0.001, and 2015–2018: *I* = 0.179; *p*≤0.001). The LISA map showed high-high clusters of AIDS mortality rates in the municipalities of the metropolitan region and in municipalities in the southeast of Pará, wherein a contraction in the cluster during the study time period was observed. A low-low cluster was also observed in all of the quadrennium’s and in all of the time periods in the Marajó and Baixo Amazonas mesoregions (Fig 3).

An ordinary least squares (OLS) regression was used to analyze the association between the age-adjusted AIDS mortality rate and SDH. The results identified per capita income and population density as the best explanatory model, but the global Moran analysis showed residual spatial dependency. Based Lagrange Multiplier robust test, the spatial lag was the best regression model to explain the influence of SDH on AIDS mortality than the spatial error (*p* = 0.02 *versus p* = 0.65, respectively) (Table 3). The model was then analyzed by GWR employing the kernel with an adaptive and fix band. The GWR with an adaptative band sowed the best adjusted regression model according the AICc criteria and residuals with no spatial dependency (Table 4).

**Table 3:** Lagrange multiplier test for detect the alternative spatial regression model for the impact of SDH on the AIDS mortality rate in Pará between 2007 and 2018.

Test	Value	p value
Lagrange Multiplier (lag)	15,366	0,0009
Robust LM (lag)	4,74	0,02
Lagrange Multiplier (error)	10,82	0,001
Robust LM (error)	0,2	0,652

**Table 4:** Comparison of OLS and GWR models for the impact of SDH on the AIDS mortality rate in Pará between 2007 and 2018.

Regression model	R^2^	AIC* / AICC**	I residual	p-value residual
OLS	0,41	640,87*	0,09	0,001
Spatial LAG	0,04	632,78*	-0,002	0,38
GWR Fix Band	0,52	631,77**	0,11	0,01
GWR Adaptative Band	0,60	626,57**	0,02	0,48

**Fig 4:**
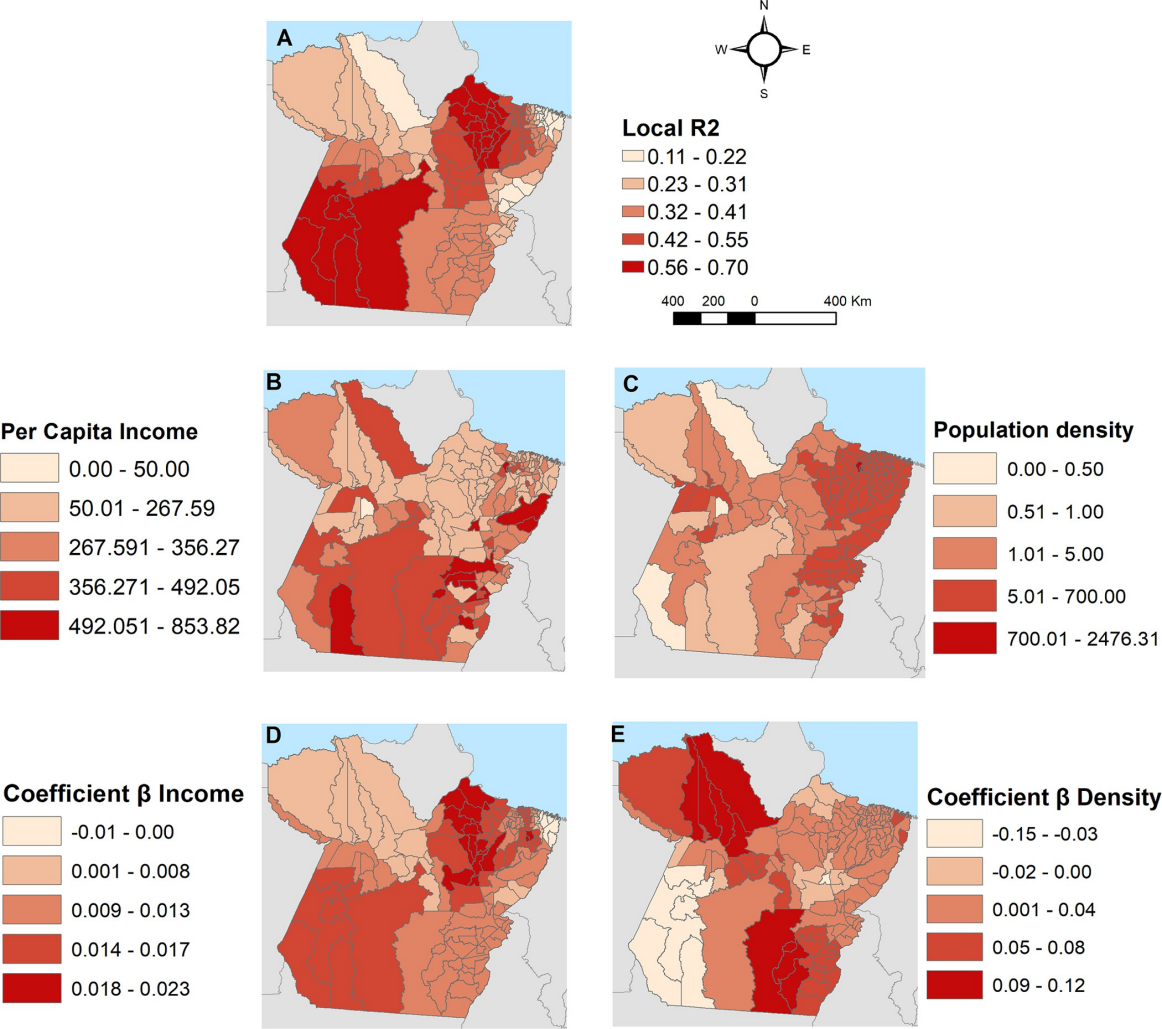
Spatial analysis by geographically weighted regression (GWR) of the social determinants of health and their relationship with AIDS mortality in the state of Pará, Brazil from 2007 to 2018. A) Local R^2^, B) Spatial distribution of per capita income, C) Spatial distribution of population density, D) β coefficients for per capita income, and E) for population density. Source: Authors.

Fig 4 illustrates the maps obtained through the GWR, with the distribution of R^2^ and coefficient β of the variables. Fig 4B and 4C illustrate the spatial distribution of the variables “per capita income” and “population demographic density”, respectively. Fig 4D shows that the risk for AIDS mortality was higher in municipalities of Southwest and Marajó regions. Meanwhile, Fig 4E shows that the risk for the AIDS mortality was higher in municipalities of Baixo Amazonas and Southeast regions.

## Discussion

The results showed that the AIDS crude mortality rate exhibited an upward trend from 2007 to 2013, followed by stabilization until 2018 and an upward (~22%) forecasted trend from 2019 to 2022. Considerable variabilities in the mortality crude rates were observed in March and May. Moreover, spatial variability was observed for the mortality rate, with the highest impact observed in the municipalities of Marajó and the southwestern area of Pará. High–high clusters were recorded in the metropolitan and southeastern regions. In addition, the GWR indicated the existence of SDH “population demographic density” and the “per capita income” associated with the geographic variation of the AIDS mortality rate in Pará.

The changes in the historical series of the mortality rate in Pará (from an increasing trend in 2007–2013 to a stabilization until 2018) can be attributed to the advances in health policies in Brazil for PLWHA that have been implemented since 2013. HIV tests have been applied in primary health care settings, thus increasing testing coverage. In the same time period, Brazil started the political “treatment for all” initiative by implementing antiretroviral therapy (ART) immediately after HIV diagnoses [32]. However, some studies have shown a lack of supplies for HIV testing in Brazilian primary health care locations [33].

In addition, although the time series seasonality analysis was not statistically significant, our results showed a greater variability in AIDS mortality in March and May. March is subsequential to the festivities of certain months in Pará, where in October the “Círio de Nazaré” occurs and in December, the end-of-year festivities occur. “Círio de Nazaré” is a Catholic event celebrated in Belém that attracts a great number of tourists (over 2 million people), with end-of-year of festivities occurring in the main procession. In addition, in May, the Feast of Saint John occurs. The cultural tradition of not stopping ART to drink alcohol (which is associated with risky behaviors) can lead people living with HIV to progress to AIDS, thus increasing mortality. A study in the United States showed that among Africans/Americans, blacks, and Latinos living with HIV, alcohol was associated with the discontinuation of ART [34].

Early diagnoses and broad implementation of ART are beneficial in infection and death control [5,35–37]. In a study conducted in the United Kingdom that analyzed the effect of late diagnoses on life expectancy, late diagnoses were identified as the main factor associated with mortality in PLWHA and increased the life expectancy of people living in locations with wide access to antiretroviral drugs and services [38]. However, in Pará, a previous study showed that 50% of people determine their serological status when afflicted with AIDS [39], which can be due to the low coverage of primary health care locations in most municipalities. Thus, an expansion of primary health care access and ART dispensation may decrease AIDS mortality in Pará. This hypothesis is consistent with the contraction of the HH cluster in southeastern Pará. In these municipalities, primary health care coverage was expanded by 180%, and 10 new MDU were created between 2006 and 2018 to primarily serve the southeastern and southwestern regions of Pará [19,40].

Our results predicted a 22% increase in the AIDS mortality rate from January 2019 to December 2022. When compared with the data shown in the Brazilian epidemiological bulletin, from 2018 to 2019, the number of deaths increased by 3.2% in Pará (2018 = 687 deaths; 2019 = 709 deaths). Although there was a reduction of 6.21% in the number of deaths due to AIDS between 2019 and 2020 (2019 = 709 deaths; 2020 = 665 deaths), this reduction could be due to the displacement of health workers from work during the COVID-19 outbreak [41]. Pará is progressing in the opposite direction of the observed decrease in AIDS mortality worldwide caused by considerable access to PLWHA, in which ART decreased AIDS mortality. From 2006 to 2017, the world experienced a reduction of 51% in AIDS mortality [42]; in Pará, the mortality rate increased by 6.5% from 2010 to 2020 [40]. It is not reasonable to expect an improvement in the AIDS epidemiological situation in a state with 144 municipalities and 28 MDU and only 7 specialized health centers (SAE) to follow-up with people living with HIV/AIDS [18].

Our GWR results identified only population density and per capita income as the determinants of this phenomenon in Pará, differing from a previous in Piauí (Brazil) that identified a more number SDH: “percentage of individuals in houses with inadequate walls”, “average of residents per household” and “% individuals in houses vulnerable to poverty without completing elementary school” [13]. This difference can be due to the fact that most of the municipalities had almost identical indicators. However, concerning per capita income and demographic density, some municipalities as those located in southern Pará and Marajó are distinguishable from each other because of their economic development in the last decade promoted by livestock, mineral extraction, and agriculture, which has attracted immigrants from several parts of Brazil. Although the Marajó’s municipalities still having a low average per capita income, their gross domestic production (GDP) are growing rapidly. For example, between 2014 and 2018 the municipality of Salva Terra and Afuá had their GDP increased by 33.72% and 13.08%, respectively. Municipalities with increased economic activities have been associated with a high risk of HIV and other sexually transmissible infections. A study among young people in Eastern and Southern Africa showed that the higher incidence of HIV was located in those areas in the midst of economic expansion [43].

In Brazil, over the past few years, there has been a gradual and continuous advance in HIV prevention with an expansion of primary health care (PHC) coverage, HIV tests, and ART. However, the implementation of these actions was not uniform due to the administrative organization of the Brazilian Unified Health System [44–46]. For example, Southern Pará had a lower coverage of primary health care and of MDU. However, after 2010, these municipalities had an improvement in urban infrastructure, with an expansion of 180% of primary health care coverage and 10 MDU locations being installed. However, the same progress was not observed in municipalities of Baixo Amazonas and of Marajó.

In addition, even PHC has expanded in Pará, the expansion was not similar among municipalities. Moreover, considering the low investment of the expansion of PHC which did not follow the municipalities population growth, it expected a low HIV testing coverage and a high rate of late HIV diagnosis. For example, in the municipalities of Marajó, between 2007 and 2018, the PHC coverage did not change as expected and they still having a low coverage in 2018 (Fig 1C and 1D). In a city of São Paulo—Brazil, although the ART decreased the AIDS mortality rate by 81.97% from 1980 to 2005, this reduction were only in the neighborhood with better life conditions [9].

Contributing even more to the AIDS mortality scenery in Pará, is the fact of both the ART and the HIV/AIDS medical follow-up protocols are still centralized and most people must travel long distances to reach these locations. This task is even more difficult when considering all of the imposed geographic barriers of the Amazonia region and the low socioeconomic condition of the population. PLWHA in Marajó must travel approximately 12 hours by boat to reach a MDU. All of these factors impact adherence to ART and can consequently increase the AIDS mortality rate. In Pakistan, the long travel distance and low economic conditions have been recognized as barriers to PLWHA ART adherence [47]. In Malawai, ART decentralization to health facilities decreased the traveling distance of individuals, thus consequently increasing ART enrollment among PLWHA [48]. In Manaus, the capital of Amazonas state, the ART decentralization to the PHC system also promoted a greater ART adherence among those PLWHA that accepted to be assisted in PHC than among those ones that continued the follow-up at the centralized place. In addition, PLWHA assisted at PHC related more satisfaction for having better access to the PHC due the shorter distances from their homes [49].

This study had some limitations. The first limitation was the use of secondary data that exhibited quality dependent on human resources. Another limitation of the ecological studies was the inability to demonstrate causality between AIDS mortality and other factors, such as stigma and sex work. In the current pandemic scenario of COVID-19, new societal behaviors and politics can also interfere in the course of the phenomenon being investigated, thereby modifying the time series course predictions. All of these relationships require further study.

### Conclusion

Our study showed that the AIDS mortality that AIDS-mortality rate in Pará increased after 2013 and stabilized until 2019. However, from 2019 to 2022 it was forecasted to increase. March and May were the months with the greater variability in the mortality rates. The spatial analysis showed a territorial expansion of the AIDS mortality with a high-high cluster comprised by the municipalities of the metropolitan region. GWR results showed that the greater risk to AIDS mortality were associated with per capita income and demographic density with the municipalities of Marajó and of southern Pará having the greater risk.

Despite the impact of advances in health policies against HIV, combined efforts are still necessary to reduce AIDS mortality in Pará. Social equity must be promoted with the expansion of PHC, MDU, and SAE, and health education must make PLWHA aware of the importance of adherence to ART and regular medical follow-ups. Additionally, the fight against HIV stigma must be encouraged to decrease AIDS mortality. The upward forecasted trend in AIDS mortality from 2019 to 2022 raises an alert and concern to health authorities for implementing focalized and efficient policies for the reduction and prevention of AIDS mortality considering regional aspects of the Northern Brazil.

## Supporting information

S1 Database (XLSX)

## Abbreviations

HIV: Human immunodeficiency virus
AIDS: Acquired immunodeficiency syndrome
SDH: Social determinants of health
ARIMA: Autoregressive model of moving average
STLF: Locally weighted polynomial regression
GWR: Geographically weighted regression
STL: Seasonal and trend decompositions by using the Loess
MDU: Medication dispensing units
SIM: Mortality information system
AIC: Akaike information criterion
AICC: Corrected Akaike information criterion
BIC: Bayesian information criterion
RMSE: Root mean square error
MAE: Mean absolute error
MAPE: Mean absolute percentage error
I: Global indicator of the spatial autocorrelation
LISA: local indicator of the spatial autocorrelation
OLS: Ordinary least squares
VIF: Variance inflation factors
LM: Lagrange Multipliers
ART: Antiretroviral therapy
GPD: Gross domestic production
PHC: Primary health care
PLWHA: People living with HIV and AIDS
SAE: Specialized health centers
